# Regulatory T-cell–intrinsic amphiregulin is dispensable for suppressive function

**DOI:** 10.1016/j.jaci.2016.01.030

**Published:** 2016-06

**Authors:** Katharine Carney, Yu-Mei (Ruby) Chang, Stephen Wilson, Clare Calnan, Pala S. Reddy, Win-Yan Chan, Timothy Gilmartin, Gilberto Hernandez, Lana Schaffer, Steven R. Head, Joanne Morley, Amanda de Mestre, Karen Affleck, Oliver A. Garden

**Affiliations:** a Department of Clinical Science and Services, Immune Regulation Laboratory, Royal Veterinary College, London, United Kingdom; b Research Support Office, Royal Veterinary College, London, United Kingdom; c GlaxoSmithKline, Platform Technology and Science R&D, Stevenage, Hertfordshire, United Kingdom; d Scripps Research Institute, La Jolla, Calif; e GlaxoSmithKline, Respiratory R&D, Stevenage, Hertfordshire, United Kingdom; f Department of Comparative Biomedical Sciences, Royal Veterinary College, North Mymms, Hatfield, Hertfordshire, United Kingdom

To the Editor:

Amphiregulin is 1 of 7 structurally and functionally related growth factors that bind the epidermal growth factor receptor (EGFR); it is present in membrane-bound, intracellular and secreted forms. Interest in a role for amphiregulin in the immune system has been growing: various cells from both the innate and adaptive arms of the immune system express the ligand, including basophils,^1^ several subsets of human T cells, and murine T_H_2 cells.^2^ Populations of tissue-specific murine CD4^+^Foxp3^+^ regulatory T (Treg) cells in the colon and striated muscle are enriched in amphiregulin during inflammation; indeed, amphiregulin plays a role in repair functions of muscle Treg cells.^3^ Treg cells transferred to amphiregulin^−/−^ RAG^−/−^ mice show suboptimal ability to prevent colitis in an adoptive transfer model, a phenotype that is rescued by amphiregulin supplementation.^4^ To further understand the potential role of amphiregulin in Treg-cell function and its role in inflammation, the abundance and functional significance of amphiregulin expressed by Treg cells from the peripheral lymphoid tissues of mice was investigated (see the Methods section and Table E1 in this article's Online Repository at www.jacionline.org). Transcriptomic analysis revealed differential expression of amphiregulin between conventional T cells (Tcons) and Treg cells (Fig 1, *A* and *B*): amphiregulin mRNA was greater than 3-fold more abundant in Treg cells than in Tcons, confirmed by quantitative RT-PCR (Fig 1, *A*, *B*, and *C*i). Heparin-binding epidermal growth factor-like growth factor (HB-EGF) also showed greater expression by Treg cells than by Tcons, as did EGFR itself (Fig 1, *A*-*C*); the remaining ligands fell below the limits of detection (data not shown).

Stimulation of the T-cell receptor (TCR) in human CD3^+^ T cells, CD4^+^ T cells,^5^ and murine T_H_2, but not T_H_1,^6^ cells, induces amphiregulin expression. In human CD4^+^ T cells, TCR engagement also augments HB-EGF expression.^5^ To determine whether a similar response occurs in murine Treg cells and Tcons (both naive and memory), we examined the kinetics of expression following TCR stimulation. A spike in amphiregulin expression was observed in all 3 populations at 3 hours (Fig 1, *D*i). Highest expression of amphiregulin mRNA at every time point was apparent in the Treg cells, followed by the mTcons and then nTcons. A significant difference between the regulatory and naive T cells was observed at every time point (Fig 1, *D*i).

There was no significant difference in HB-EGF expression in Treg cells over the time course, remaining higher than both Tcon populations at each point except 72 hours (Fig 1, *D*ii). In both subsets of Tcons, EGFR mRNA expression displayed a sustained increase from the first to the final time point (Fig 1, *D*iii). To determine whether the differential expression of the mRNA encoding amphiregulin and HB-EGF reflected differences in protein abundance, secreted amphiregulin and HB-EGF were quantified in culture supernatants. Amphiregulin secreted from nTcons remained below the assay detection limit of 7.8 pg/mL. The concentration of amphiregulin in the Treg-cell supernatants exceeded that of mTcons from 48 hours onward (Fig 1, *E*). Concentrations of HB-EGF were below the lower limit of detection in all supernatant samples (data not shown).

To establish whether the higher amphiregulin expression observed in Treg cells reflected a role in their suppressive function, bead-based assays using syngeneic and cross-over cocultures of Treg cells and Tcons from wild-type (WT) and amphiregulin knock-out (KO) mice were performed, following confirmation of their genotype (Fig 2, *A*). An inhibitory effect was apparent at all Treg-cell:Tcon ratios, with no difference between WT and KO cells; in particular, KO:KO cocultures, with neither an intrinsic nor an extrinsic source of amphiregulin in the cultures, showed potent suppression (Fig 2, *B* and *C*). Moreover, recombinant amphiregulin added to cultures of T cells *in vitro* impacted neither their proliferation nor their apoptosis, showing no evidence of an inhibitory role (data not shown). The suppression assays were repeated using WT accessory cells and soluble anti-CD3 mAb to activate cocultures of WT and KO Treg cells and Tcons, to establish whether Treg-cell suppression *via* antigen-presenting cells requires an intrinsic source of amphiregulin. Once again, there was no difference in suppression between any of the coculture permutations.

A regulatory role for amphiregulin is not intuitive, as the ligand has a number of proinflammatory associations including its induction of the cytokines IL-1α and IL-1β, which positively feed back to promote amphiregulin secretion.^7^ However, other studies have found a role for the ligand in the function of various subsets of murine Treg cells.^2^ Augmentation of human Treg-cell–mediated suppression of CD4^+^ Tcons *in vitro* in response to recombinant amphiregulin was observed by Zaiss et al.^4^ In the same study, enhanced activity of murine Treg cells was also apparent in response to recombinant amphiregulin, but CD8^+^ T cells were used as the responder population in these experiments, with no information on the inhibitory effect on murine CD4^+^ Tcons. The ligand also enhances Treg-cell suppression of the antiviral effects of CD8^+^ T cells *in vivo*.^8^ Amphiregulin may therefore augment Treg-cells' suppressive activity in a CD8^+^ T-cell–specific manner, but whether amphiregulin itself represents a mechanism of suppression by Treg cells in certain cellular contexts remains unknown.

Our data demonstrate that despite being more abundantly expressed by Treg cells than by Tcons, Treg-cell–intrinsic amphiregulin is not required for suppressive function *in vitro* because functional redundancy was demonstrated in 2 well-established *in vitro* assays of suppression, suggesting that amphiregulin cannot be considered a core regulatory mechanism of these cells. Rather, we speculate that amphiregulin is involved in the tissue-reparative effects of Treg cells, in a manner that is independent of regulatory function. This viewpoint is supported by recently published work that complements our study^2^, ^9^ and extends our current insight into the complex, multifaceted roles of amphiregulin in health and disease.

## Figures and Tables

**Fig 1:**
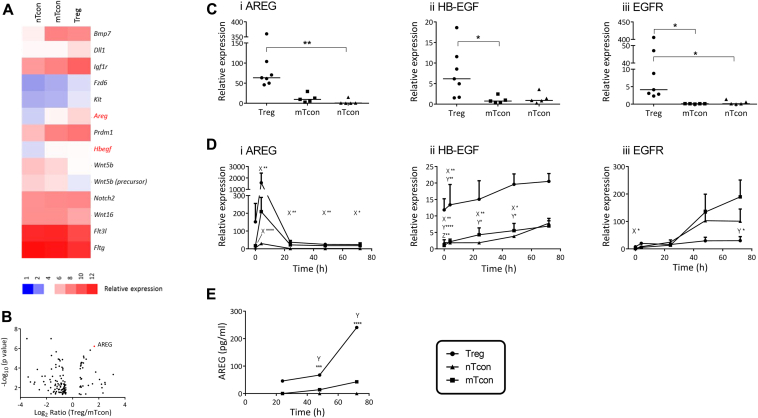
Treg cells show an amphiregulin^high^ phenotype. Peripheral Treg cells and memory (m) and naive (n) Tcons selected from naive WT mice revealed greater amphiregulin and HB-EGF transcript abundance by microarray analysis (**A**: heat map; **B**: volcano plot) and quantitative RT-PCR assays (**C**; n ≥ 5), both *ex vivo* and following polyclonal stimulation (**D**, n = 3; **E**, n ≥ 2). *AREG*, Amphiregulin. Key: Statistical significance between X = Treg cells/nTcons, Y = Treg cells/mTcons, and Z = nTcons/mTcons (*P < .05, **P < .01, ***P < .001, ****P < .0001).

**Fig 2:**
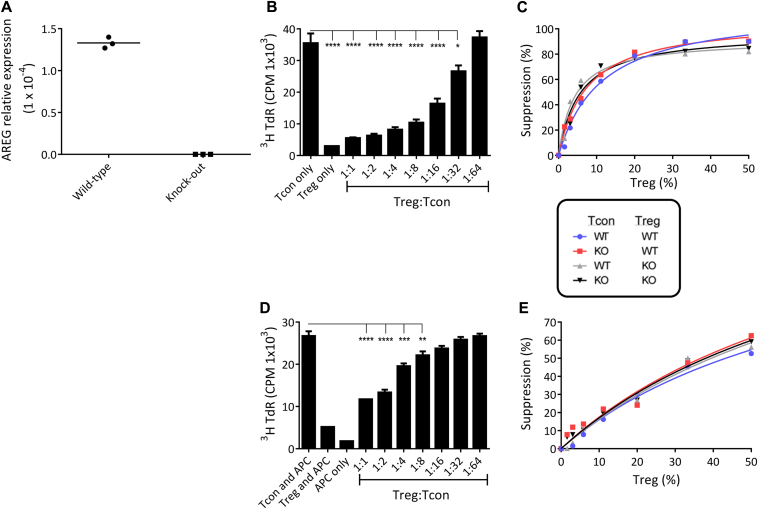
Treg-cell–intrinsic amphiregulin is not required for suppressive function *in vitro*. **A,** Confirmation of genotype by quantitative RT-PCR. Cocultured Tcons and Treg cells were stimulated with (**B** and **C**) anti-CD3/CD28-coated Dynabeads or (**D** and **E**) soluble anti-CD3 with WT antigen-presenting cells (APCs). *B* and *D*, Counts per minute (CPM) in 1 representative experiment (*P = .011, **P = .0065, ***P = .0002, ****P < .0001); *C* and *E*, proportional suppression of CPM. *A*, n = 1; *B* and *C*, n = 3; *D* and *E*, n = 1. *AREG*, Amphiregulin.

## Supplementary data

Methods

Table E1
