# Supplementary material for: Regulatory T-cell–intrinsic amphiregulin is dispensable for suppressive function
Source: J Allergy Clin Immunol. 2016 Jun;137(6):1907–9. doi: 10.1016/j.jaci.2016.01.030 (PMC4889774; doi:10.1016/j.jaci.2016.01.030)
Supplement: Methods [file mmc1.docx]

Methods

Mice

AREG^tm1Dle/Mmnc^ mice on a C57BL/6J x 129S1/SvJ background were obtained from the Mutant Mouse Regional Resource Centre (MMRRC; University Of North Carolina, Chapel Hill, USA). The homozygous null mutant mice were bred onto a C57BL/6J background (obtained from Charles River Laboratories, UK) in the GlaxoSmithKline (GSK) Laboratory Animal Sciences department (Stevenage, UK), confirming zygosity by PCR. Following multiple generations of backcrossing to C57BL/6J, the genetic background of the strain was confirmed by single-nucleotide polymorphism analysis using the MAX-BAX^®^ platform (Charles River Laboratories) to be greater than 98% C57BL/6J. To generate animals for study, heterozygous AREG^tm1Dle/Mmnc^ breeding pairs were mated to generate homozygous AREG knock-out and wild-type litter-mate controls. All animal procedures were reviewed and approved by the GSK Animal Care and Use Committee, and were performed in AAALAC accredited facilities in accordance with the Animals (Scientific Procedures) Act 1986 and the GSK Policy on the Care, Welfare and Treatment of Laboratory Animals. Mice were genotyped, weaned, and separated based on sex at three weeks of age, then multiply-housed in open-rack caging at an ambient temperature 19–21°C and humidity 45-55% maintained on a 06:00h:20:00h light–dark cycle, with free access to maintenance diet (LabDiet^®^ custom 5LFL) and animal-grade drinking water. GSK animal facilities are maintained at Specific Pathogen Free status, with routine health monitoring carried out to FELASA recommendations.

Cell isolation

Following Schedule 1 euthanasia by exposure to CO_2_ gas in a rising concentration and confirmation of death by cervical dislocation, lymph nodes and spleens were harvested from mice, macerated and washed with RPMI-1640. Erythrocytes were lysed using ammonium chloride-potassium bicarbonate buffer. Fluorescence-activated cell sorting (FACS^™^) was employed to isolate Treg (CD4^+^CD25^+^CD45RB^low^), naïve (n) Tcon (CD4^+^CD25^-^CD45RB^high^) and memory (m) Tcon (CD4^+^CD25^-^CD45RB^low^) populations. Cells were stained with antibodies against CD4, CD45RB and CD25 (all eBioscience, Hatfield, UK), before FACS^™^ on a MoFlo^™^ cell sorter (Beckman Coulter, High Wycombe, UK).

Microarray

An RNeasy mini kit (QIAGEN, Manchester, UK) was used for total RNA extraction, following the manufacturer’s protocol. A microarray assay was performed using the Glyco-gene Chip v4, containing 1,127 probe sets targeting glycosylation-related transcripts including genes encoding proteins that are either heavily glycosylated or involved in glycosylation, as well as 119 probe sets targeting reference (‘housekeeping’) transcripts (see Gene Lists). Robust Multichip Average (RMA) software was used to convert intensity to expression values^1^ and Cluster software was used for hierarchical clustering. Analysis of variance performed by BRB Array Tools was used to identify genes on the arrays showing significant differences at or below a 20% false discovery rate, focusing on changes greater than 1.4-fold. Heat maps were generated with JavaTreeViewer. All transcriptomic data have been uploaded to the National Institutes of Health Gene Expression Omnibus database, accession number GSE31921 (<http://www.ncbi.nlm.nih.gov/geo/query/acc.cgi?acc=GSE31921>).

Quantitative reverse transcriptase (RT)-PCR

Gene Expression Assays (Primer Design, Southampton, UK) containing double-dye probes with non-complementary primers specific to the target transcripts were used with Precision OneStep^™^ qRT-PCR MasterMix (Primer Design). Reactions with >38 cycle quantification (C_q_) were discounted from analysis. Samples were run in triplicate.

For each transcript standard curves were generated, plotting the C_q_ values against the dilution factor and using the slope to calculate amplification efficiency. These values were used to calculate the abundance of transcript in mRNA from cells of interest, relative to a control population of unselected CD4^+^ T cells at time 0, using Pfaffl’s model^2^.

Six candidate reference genes were screened for stability across the populations of interest using the geNorm system. *Actb* and *Gapdh* were selected.

Cell culture

Cells were cultured in round bottom 96-well plates in RPMI-1640 supplemented with 2mM L-glutamine, 100U/ml penicillin, 100µg/ml streptomycin, 10mM 4-(2-hydroxyethyl) piperazine-1-ethanesulfonic and 10% v/v fetal calf serum (complete medium). All culture conditions, including those of suppression assays, were set up in triplicate.

ELISA

Secreted AREG was measured in culture supernatants by means of an ELISA DuoSet kit for murine AREG (R&D Systems, Minnesota, USA); complete medium at time 0 contained no detectable AREG (data not shown). Owing to a lack of a commercially available murine heparin-binding EGF-like factor (HB-EGF) ELISA, a human HB-EGF ELISA kit (R&D Systems) was used, generating a standard curve with murine recombinant HB-EGF (ProSpec, East Brunswick, New Jersey, USA). The assay detection limit for murine HB-EGF was 125pg/ml. All samples were run in triplicate.

Suppression assays

Tregs and nTcons were co-cultured in complete medium. Proliferation was induced by means of anti-CD3/CD28 mAb-coated Dynabeads^®^ (Invitrogen) or mitomycin C-treated (25µg/mL) splenocytes in the presence of 250ng/ml anti-CD3 mAb for three days. To measure proliferation, cells were pulsed with tritiated thymidine (^3^H-TdR) for 18 hours before measurement of ^3^H-TdR incorporation with a cell harvester and liquid scintillation counter (Wallac Trilux, Perkin Elmer, UK).

Statistical tests

Statistical significance of transcript expression (Fig 1) was determined by the Kruskal-Wallis test followed by the Dunn’s multiple comparisons test; a linear mixed model followed by Holm’s Bonferroni correction was used to obtain post-hoc *p* value cut-off points. Horizontal bars in the figures represent median values, while error bars represent standard errors of the mean. Statistical significance of suppression (Fig 2) was determined using one-way ANOVA followed by the Holm Sidak’s multiple comparison test. Significance was assumed when *p*<0.05.

References

1. Bolstad BM, Irizarry RA, Astrand M, Speed TP. A comparison of normalization methods for high density oligonucleotide array data based on variance and bias. Bioinformatics 2003;19:185-93.

2. Pfaffl MW. A new mathematical model for relative quantification in real-time RT-PCR. Nucleic Acids Res 2001;29:e45.
